# At‐sea distribution and habitat use in king penguins at sub‐Antarctic Marion Island

**DOI:** 10.1002/ece3.2833

**Published:** 2017-04-22

**Authors:** Pierre Pistorius, Mark Hindell, Robert Crawford, Azwianewi Makhado, Bruce Dyer, Ryan Reisinger

**Affiliations:** ^1^ Department of ZoologyDST/NRF Centre of Excellence at the Percy FitzPatrick Institute for African OrnithologyNelson Mandela Metropolitan UniversityPort ElizabethSouth Africa; ^2^ Institute for Marine and Antarctic StudiesUniversity of TasmaniaHobartTasAustralia; ^3^ Oceans and CoastsDepartment of Environmental AffairsCape TownSouth Africa; ^4^ Animal Demography UnitUniversity of Cape TownRondeboschSouth Africa; ^5^ DST/NRF Centre of Excellence at the Percy FitzPatrick Institute for African OrnithologyUniversity of Cape TownRondeboschSouth Africa

**Keywords:** foraging, habitat selection, movement, seabirds, Southern Ocean

## Abstract

King penguins make up the bulk of avian biomass on a number of sub‐Antarctic islands where they have a large functional effect on terrestrial and marine ecosystems. The same applies at Marion Island where a substantial proportion of the world population breeds. In spite of their obvious ecological importance, the at‐sea distribution and behavior of this population has until recently remained entirely unknown. In addressing this information deficiency, we deployed satellite‐linked tracking instruments on 15 adult king penguins over 2 years, April 2008 and 2013, to study their post‐guard foraging distribution and habitat preferences. Uniquely among adult king penguins, individuals by and large headed out against the prevailing Antarctic Circumpolar Current, foraging to the west and southwest of the island. On average, individuals ventured a maximum distance of 1,600 km from the colony, with three individuals foraging close to, or beyond, 3,500 km west of the colony. Birds were mostly foraging south of the Antarctic Polar Front and north of the southern boundary of the Antarctic Circumpolar Current. Habitat preferences were assessed using boosted regression tree models which indicated sea surface temperate, depth, and chorophyll a concentration to be the most important predictors of habitat selection. Interestingly, king penguins rapidly transited the eddy‐rich area to the west of Marion Island, associated with the Southwest Indian Ocean Ridge, which has been shown to be important for foraging in other marine top predators. In accordance with this, the king penguins generally avoided areas with high eddy kinetic energy. The results from this first study into the behavioral ecology and at‐sea distribution of king penguins at Marion Island contribute to our broader understanding of this species.

## 1 Introduction

Land‐breeding marine top predators, particularly seabirds and seals, are abundant in the sub‐Antarctic region where they constitute an important ecological element. Population numbers in most of these species have, however, been particularly dynamic over the past few decades, with this potentially linked to large‐scale environmental changes in the Southern Ocean (Weimerskirch, Inchausti, Guinet, & Barbraud, 2003). Such changes are generally associated with prey resource availability and distribution (Crawford, Dyer, Upfold, & Makhado, 2014; Crawford et al., 2010; Pistorius et al., 2004).

Marine top predators generally feed on patchily distributed prey resources over a range of spatial scales. Locating such prey is probably the most important challenge faced by these animals as it impacts both their survival and fecundity. It is therefore reasonable to assume that there is strong selective pressure in marine top predators to optimize prey searching behavior and to use appropriate oceanographic features to find profitable prey patches. A number of studies have demonstrated the importance of mesoscale features, such as eddies, to foraging marine top predators because of their importance as centers of biological production and their consequently high prey concentrations (Cotté, Park, Guinet, & Bost, 2007; Ream, Sterling, & Loughlin, 2005; Strass et al., 2002). For example, grey‐headed albatrosses (*Thalassarche chrysostoma*) tracked from Marion Island in the southwest Indian Ocean targeted mesoscale eddies associated with the Southwest Indian Ocean Ridge to the west of the island (Nel et al., 2001), and southern elephant seals (*Mirounga leonina*) demonstrated relatively high dive frequency in an eddy field also to the southwest of the island (Massie et al., 2016).

The king penguin (*Aptenodytes patagonicus*) is the second largest penguin species and is a deep‐diving, far‐ranging species and one of the primary avian consumers in the Southern Ocean (Bost et al., 1997; Charrassin & Bost, 2001; Gurney, Pakhomov, & Christensen, 2014). It has a circumpolar distribution, breeding on islands in close proximity to the Antarctic Polar Front (APF) where mixing and upwelling creates productive waters. With the exception of the Falkland Islands (Baylis et al., 2015; Pütz, 2002), where birds target the sub‐Antarctic region in summer, the APF in autumn, and areas north of the islands along the Patagonian slope in winter, king penguins breeding elsewhere have been shown to feed close to the APF during summer and forage further afield in Antarctic waters during winter (Bost, 2004; Charrassin & Bost, 2001; Pütz, 2002; Trathan et al., 2008). King penguins have an unusual asynchronous breeding cycle, lasting about 13 months, which includes a winter chick‐fasting period when adults undertake long foraging trips probably due to food limitation in their summer foraging zones in winter (Bost, 2004; Descamps, Gauthier‐Clerc, Gendner, & le Maho, 2002). Chicks hatch in early summer and only fledge the following spring. The king penguin population at the Prince Edward Islands archipelago numbers roughly 170,000 breeding pairs for Marion Island and 5,000 for neighboring Prince Edward Island, representing some 13% of the world population (Crawford et al., 2010).

In this study, for the first time we described the at‐sea distribution of king penguins from Marion Island during the latter part of the chick‐rearing period. Using a presence–availability approach (Aarts, Mackenzie, McConnell, Fedak, & Matthiopoulos, 2008), we furthermore modeled preferred at‐sea habitat and the relationship between a range of environmental variables and a measure of foraging behavior. Two hydrographic frontal systems straddle the Prince Edward Islands—the Polar Front and the sub‐Antarctic Front—and we furthermore investigated the importance of these frontal zones for foraging king penguins. We were also interested in determining whether penguins preferentially associate with mesoscale oceanographic features (indexed by sea surface temperature and sea surface height) and strong currents as has been observed elsewhere (Baylis et al., 2015; Cotté et al., 2007).

## 2 Methods

### 2.1 Study site

**Figure 1:**
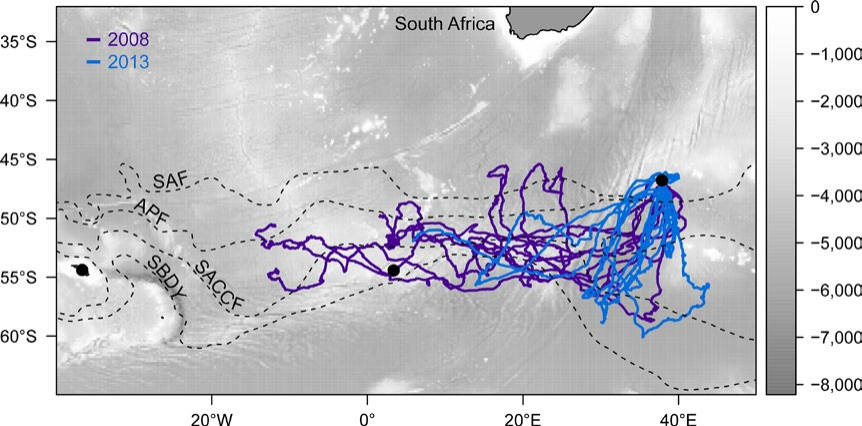
Map of the tracks of 15 adult king penguins from Marion Island in 2008 (purple) and 2013 (blue). Also indicated are the bathymetry (background, m) and the mean positions (Orsi et al. 1995) of major oceanographic fronts (dashed lines): the Subantarctic Front (SAF), Antarctic Polar Front (APF), southern Antarctic Circumpolar Current Front (SACCF), and southern boundary of the Antarctic Circumpolar Current (SBDY). Black points indicate (from west to east) South Georgia, Bouvet Island, and Marion Island

Sub‐Antarctic Marion Island (46°54′S, 37°45′E) lies approximately 2,200 km southeast of Cape Town, South Africa (Figure 1). It is about 290 km^2^ in area, with a coastline of approximately 72 km in circumference dominated by cliff faces. King penguins primarily occupy beaches on the eastern side of the island, the exception being a breeding colony on the south coast at Goodhope Bay. Field work was conducted at Kildalkey Bay (hosting about half of the Marion Island population) and Archway Bay, about 8 km north of Kildalkey, where a relatively small colony of about 2,000 pairs breeds.

### 2.2 Deployment of instruments

Seven and eight platform transmitter terminals (PTTs) were deployed respectively between 3 and 7 April 2008 and during 27 and 28 April 2013 on chick‐rearing adult king penguins at Marion Island. Penguins were captured after individuals were observed feeding their chicks and were physically restrained for instrument deployment. Telonics ST‐10 satellite transmitters were used in 2008, while Sirtrack KiwiSat K2G (Sirtrack Ltd., New Zealand) units were used for 2013 deployments. Instruments were hydrodynamically streamlined weighing 270 and 208 g, respectively, i.e., approximately 2% of body weight, and fitted to the back along the midline with the antennae positioned posteriorly. For deployment of instruments, one strip of a velcro (velcro ^®^brand) hook and loop fastener was glued to the bottom of the instrument and the other strip to the back of the penguin using cyanoacrylate glue (Loctite^®^401). After approximately one minute (when dry) the instrument was pushed down onto the back of the bird with some additional glue between the opposing velcro strips. Two small cable ties were then inserted through the feathers underneath the instrument and tightened. Handling time per bird was no more than 15 min.

### 2.3 Data analyses

All analyses were conducted in the R environment (R Core Team, 2016). State‐space models are increasingly being used to model animal movement (Jonsen et al., 2013) and these were fitted to our data using the *bsam* package in R (Jonsen et al., 2013), estimating locations at 2‐hr intervals. The state‐space model implemented fits a first‐difference correlated random walk movement model to the Argos‐derived tracking data. At the same time, the model accounts for the errors associated with the location estimates in the tracking data (Jonsen, Mills Fleming, & Myers, 2005). We investigated habitat selection using a presence–pseudoabsence approach. Using the *adehabitat* package (Calenge, 2006) we simulated 20 correlated random walk tracks for every track estimated using the state‐space model (hereafter, “estimated tracks”). The scaling parameter for the step length (*h*) was measured from the corresponding estimated tracks and turning angles at each step were drawn from a wrapped normal distribution with a concentration parameter (*rho*) estimated from the corresponding estimated tracks (*wle* package, Agostinelli & SLATEC Common Mathematical Library (1995)). Simulated positions further than the maximum distance from the island observed in the estimated tracks were discarded, as were positions on land and those where sea ice concentration was >90%.

Longer residence time is associated with periods of longer prey searching in king penguins and a greater number of foraging dives (Péron, Weimerskirch, & Bost, 2012). Time spent per grid cell was thus used as a proxy for foraging effort, as it is likely to correlate with areas where penguins spent more time foraging. Mean time spent per 50 km grid cell (sum of time spent divided by number of individuals) was calculated in each year using the *trip* package (Sumner, 2013). The cell size was selected such that cells aggregated several penguin location estimates each, and were larger than most of the environmental covariate cells (see below), but still small enough to describe penguin movement at a biophysically relevant mesoscale.

To investigate the influence of physical and biological oceanographic features on the movements of the penguins, we compiled two static environmental covariates (bathymetry and bathymetry gradient), eight dynamic covariates (sea surface temperature, sea surface temperature gradient, sea surface height anomaly, sea surface height gradient, chorophyll a concentration, net primary production, eddy kinetic energy, and sea ice concentration) and one climatology (mixed layer depth) using the *raster* (Hijmans, 2015) and *raadtools* (Sumner, 2015) packages, and the data sources listed in Supplementary Table S1. Dynamic covariates were obtained daily to monthly for the entire tracking period in each year, and were averaged over the tracking period with further interpolation (where necessary) by ordinary Kriging (Nychka, Hammerling, Sain, Lenssen, & Nychka, 2016). The monthly climatology for mixed layer depth was averaged over tracking months. These broadscale habitat variables are proxies for ocean processes and features that influence the aggregation of prey (e.g., fronts and mesoscale ocean features) and are typically associated with the foraging behavior of penguins (Péron et al., 2012). Values for each covariate were then extracted for each real or simulated penguin location estimate or for each 50 km grid cell in which there were penguin location estimates.

Boosted regression trees (BRTs) (Friedman, 2001) were used firstly to model the habitat used by king penguins (the presence–pseudoabsence approach) and secondly to model the relationship between the environmental covariates and time spent per cell. BRTs are an ensemble learning approach where many (typically 100s–1,000s) simple regression trees are fitted to the data iteratively to produce a single model; at each step a tree is fitted which best reduces residual deviance (Elith, Leathwick, & Hastie, 2008). BRTs flexibly fit nonlinear relationships and do not require prior data transformation or outliers to be removed (Elith et al., 2008). Only half the data are used to fit the tree at each step, to reduce the model variance, and the contribution of each tree is reduced by a learning rate or shrinkage parameter, given below. Tree complexity (maximum interaction depth between predictors) was set at 5, and the number of trees to fit in the final model was determined by assessing deviance reduction during 10‐fold cross‐validation of models containing 100–20,000 trees (Elith et al., 2008). BRTs were fitted in the *dismo* (Hijmans, Phillips, Leathwick, Elith, & Hijmans, 2016) and *gbm* (Ridgeway, 2010) packages. Where correlation between predictors was >0.7 (Dormann et al., 2013), the predictor with the higher average correlation to other predictors was excluded from models (net primary production, sea surface height gradient, and current). In order to characterize habitat used we considered location estimates as a binomial response, taking the value 1 where a location was from an estimated track and 0 where a location was from a simulated track. We fitted 13,850 trees at a learning rate of 0.05. For foraging behavior, the average time spent per cell (hours) was considered a Gaussian response. We fitted 3,150 trees at a learning rate of 0.05. Models were evaluated through stratified 10‐fold cross‐validation in the *caret* package (Kuhn, 2016) using area under the receiver operating characteristic curve (AUC) for the habitat selection model and root mean squared error (RMSE) and the coefficient of determination (*R* ^2^) for the time‐spent model.

## 3 Results

**Figure 2:**
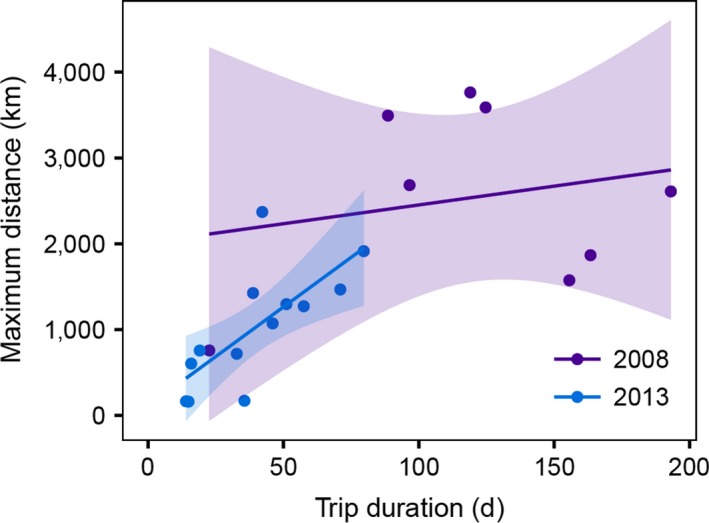
The relationship between track duration and the maximum distance of penguins from Marion Island in 2008 (purple) and 2013 (blue). Linear regressions were fitted and shaded areas indicate 95% confidence bands

We tracked 15 birds, which made 21 trips over the autumn and winter in two years (Table 1). Eight foraging trips were recorded by the seven individuals from the 2008 deployments between April and December and 13 trips by eight individuals between April and July 2013. A total of 12,347 at‐sea locations were received. The penguins tracked in 2008 made fewer, longer, and more distant trips than those in 2013. These differences were pronounced, with the mean tracking duration in 2008 (120 ± 52 days) three times longer than in 2013 (39.9 ± 21.2 days) (Welch's *t *=* *4.11, *df* = 8.41, *p *=* *.003). The 2008 durations will be underestimates as five of the animals’ tags failed before they returned to Marion Island (Table 1). Maximum distance from Marion Island was also significantly different between the 2 years. The birds in 2008 travelled an average (±*SD*) of 2,542 ± 1,075 km (again potentially an underestimate) compared to 1,030 ± 686 km in 2013 (Welch's *t *=* *3.56, *df* = 10.56, *p *=* *.005). Overall, tracking duration was positively related to maximum distance from Marion Island (Figure 2). This was pronounced among the 2013 birds; a linear mixed‐effects model (*lme4* package, Bates, Mächler, Bolker, & Walker, 2015) with individual as a random effect showed a significant relationship between tracking duration and maximum distance (slope = 18.55, *p *=* *.005). There was little pattern among the 2008 birds in this regard (slope = 4.38, *p *=* *.538) and when the influential trip lasting only 22.6 days was removed the relationship was negative, but still nonsignificant (slope = −13.27, *p *=* *.170).

**Table 1:** Trip characteristics of the 15 king penguins from Marion Island tracked in 2008 and 2013

Year	ID	Trip	Complete trip	Start (GMT)	End (GMT)	Track duration (d)	Maximum distance (km)
2008	57331	1	No	2008/04/22 22:27	2008/10/03 8:27	163.4	1,866
2008	57332	1	Yes	2008/04/20 18:27	2008/10/30 20:27	193.1	2,610
2008	57335	1	Yes	2008/04/04 4:22	2008/04/26 18:22	22.6	757
2008	57335	2	No	2008/04/26 22:22	2008/08/23 22:22	119.0	3,763
2008	57339	1	No	2008/04/05 1:39	2008/08/07 17:39	124.7	3,589
2008	57345	1	No	2008/04/12 0:28	2008/07/09 14:28	88.6	3,493
2008	57346	1	No	2008/04/11 12:21	2008/09/14 2:21	155.6	1,574
2008	57349	1	No	2008/04/18 2:21	2008/07/23 16:21	96.6	2,683
	Mean					120.1 ± 52.3	2,542 ± 1,075
2013	119305	1	Yes	2013/05/01 1:55	2013/05/16 23:55	15.9	604
2013	119305	2	Yes	2013/05/24 5:55	2013/07/09 5:55	46.0	1,072
2013	119306	1	Yes	2013/05/01 5:54	2013/06/02 23:54	32.8	718
2013	119307	1	Yes	2013/04/28 5:54	2013/06/05 23:54	38.8	1,426
2013	119307	2	No	2013/06/19 1:54	2013/07/31 5:54	42.2	2,370
2013	119308	1	Yes	2013/05/01 3:51	2013/05/15 3:51	14.0	165
2013	119308	2	Yes	2013/05/20 3:51	2013/06/04 1:51	14.9	161
2013	119308	3	No	2013/06/13 7:51	2013/07/18 21:51	35.6	171
2013	119309	1	Yes	2013/04/29 17:55	2013/05/18 19:55	19.1	756
2013	119309	2	Yes	2013/05/31 7:55	2013/07/21 9:55	51.1	1,297
2013	119310	1	Yes	2013/05/11 10:49	2013/07/30 2:49	79.7	1,914
2013	119311	1	Yes	2013/05/07 4:45	2013/07/03 16:45	57.5	1,272
2013	119312	1	No	2013/05/21 6:50	2013/07/31 6:50	71.0	1,467
	Mean					39.9 ± 21.2	1,030 ± 686

**Figure 3:**
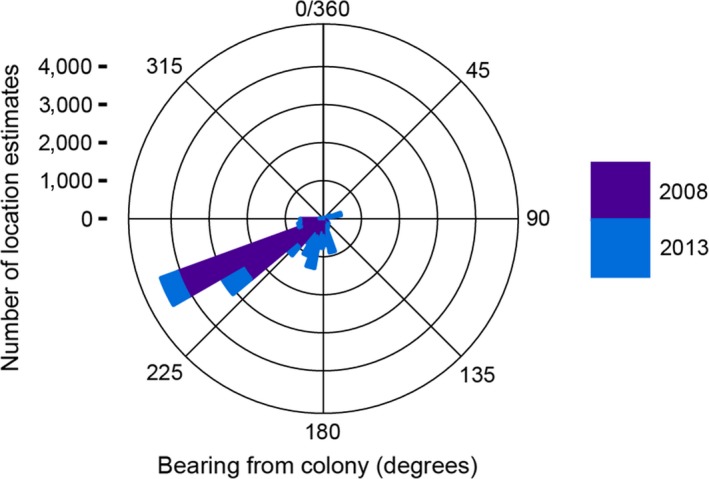
Stacked histogram of bearing from colony (°) to at‐sea locations for 15 king penguins tracked in 2008 (purple) and 2013 (blue)

There was a remarkable similarity between birds in the initial dispersal pattern, with birds generally heading out in a south or southwesterly direction before changing to a more westerly bearing, against the prevailing Antarctic Circumpolar Current (Figure 3), although birds in 2013 showed more southerly bearings (Figures 1 and 3). Some birds approximated 60°S when south of 50°S but most birds deviated course in a westerly direction once south of this latitude and the majority of locations were between the Antarctic Polar Front and the southern boundary of the Antarctic Circumpolar Current (median latitude = 52.2°S, interquartile range (IQR) = 54.3°S–50.1°S). A number of foraging trips extended well into the Atlantic Ocean and up to about 13°W (Figure 1). Whereas some tracks seemed to enter the pack ice based on our seasonal climatology, only one individual actually appeared to encounter ice. Based on matched daily sea ice concentration (not shown), individual 577331 was present in a grid cell with 3.5% sea ice concentration on 2008/10/01.

**Figure 4:**
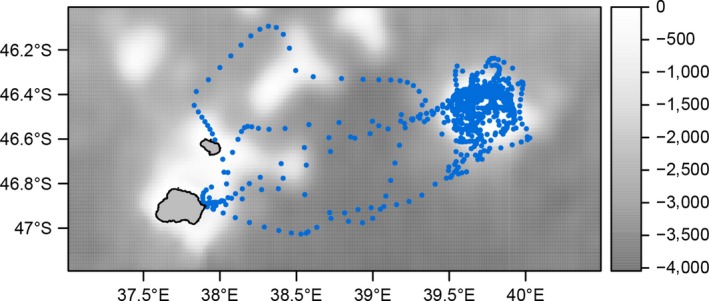
Positions (blue points) of king penguin individual 119308 which made three trips to the Galieni Bank to the northeast of Marion Island in 2013. Background shading shows bathymetry (m)

A notable exception to the above pattern was individual 119308, which made three foraging trips to the Galieni Bank ~140 km northeast of Marion Island (Figure 4).

**Figure 5:**
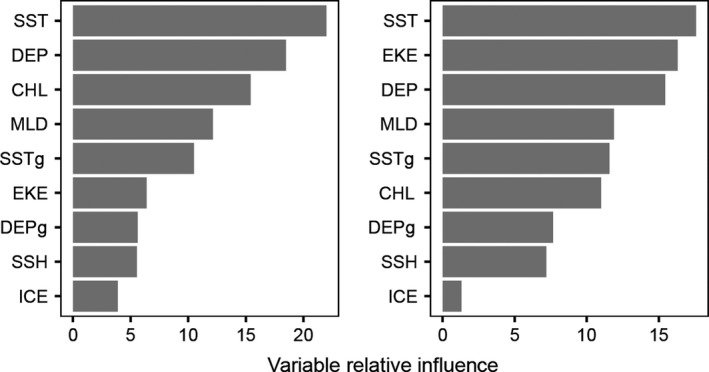
Relative influence of environmental covariates used to model habitat selection (left panel) and time spent per grid cell (right panel). SST, sea surface temperature; DEP, depth; CHL, chorophyll a concentration; MLD, mixed layer depth; SSTg, sea surface temperature gradient; EKE, eddy kinetic energy; DEPg, depth gradient; SSH, sea surface height anomaly; ICE, sea ice concentration

**Figure 6:**
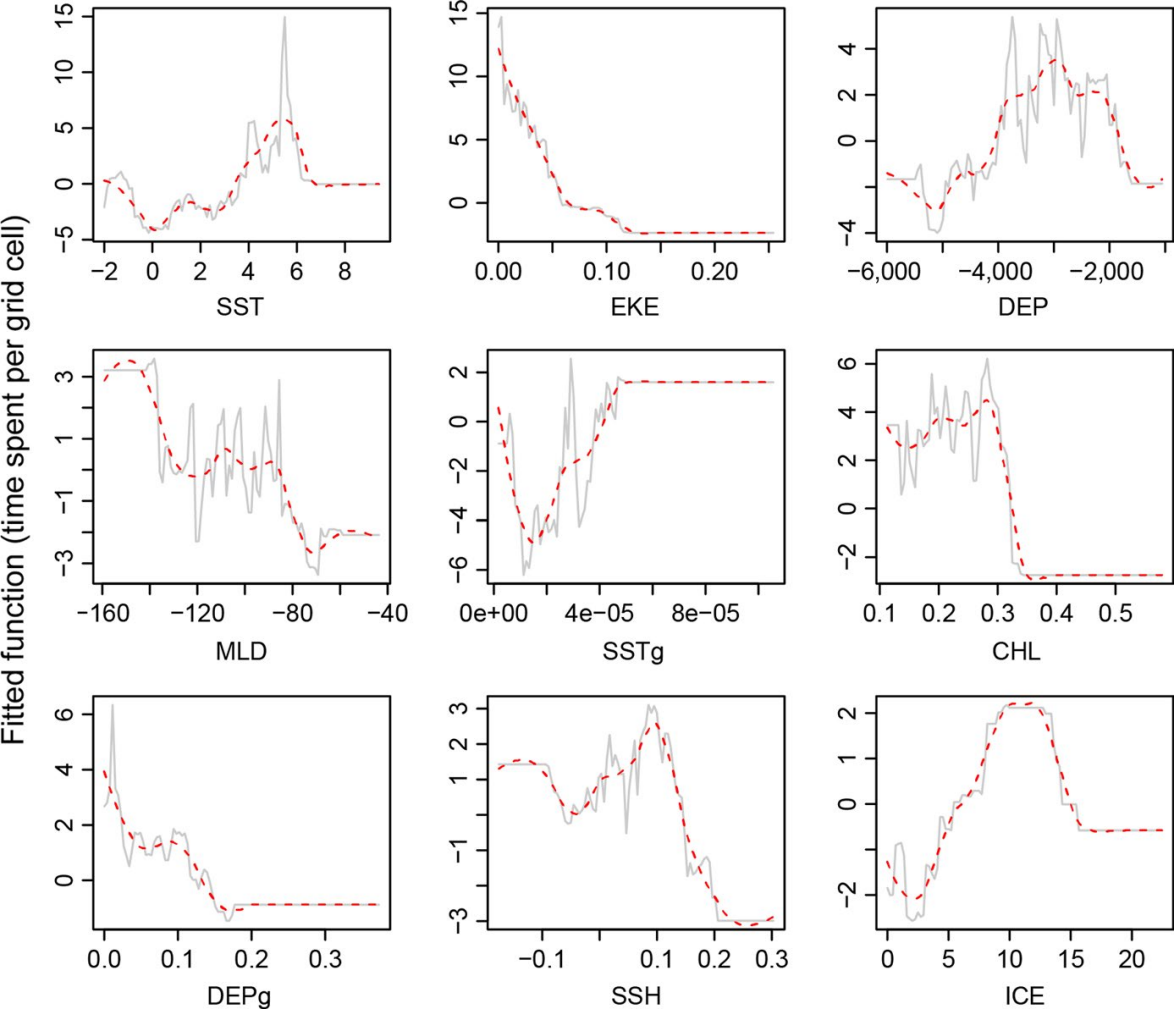
Fitted functions of nine environmental covariates explaining time spent per grid cell. Dashed red lines show smoothed functions of the fitted functions, and plots are ordered by importance of the covariates in the final boosted regression tree model. SST, sea surface temperature (°C); DEP, depth (m); CHL, chorophyll a concentration (mg/m^3^); MLD, mixed layer depth (m); SSTg, sea surface temperature gradient (radians); EKE, eddy kinetic energy (cm^2^/s^2^); DEPg, depth gradient (radians); SSH, sea surface height anomaly (m); ICE, sea ice concentration (%)

**Figure 7:**
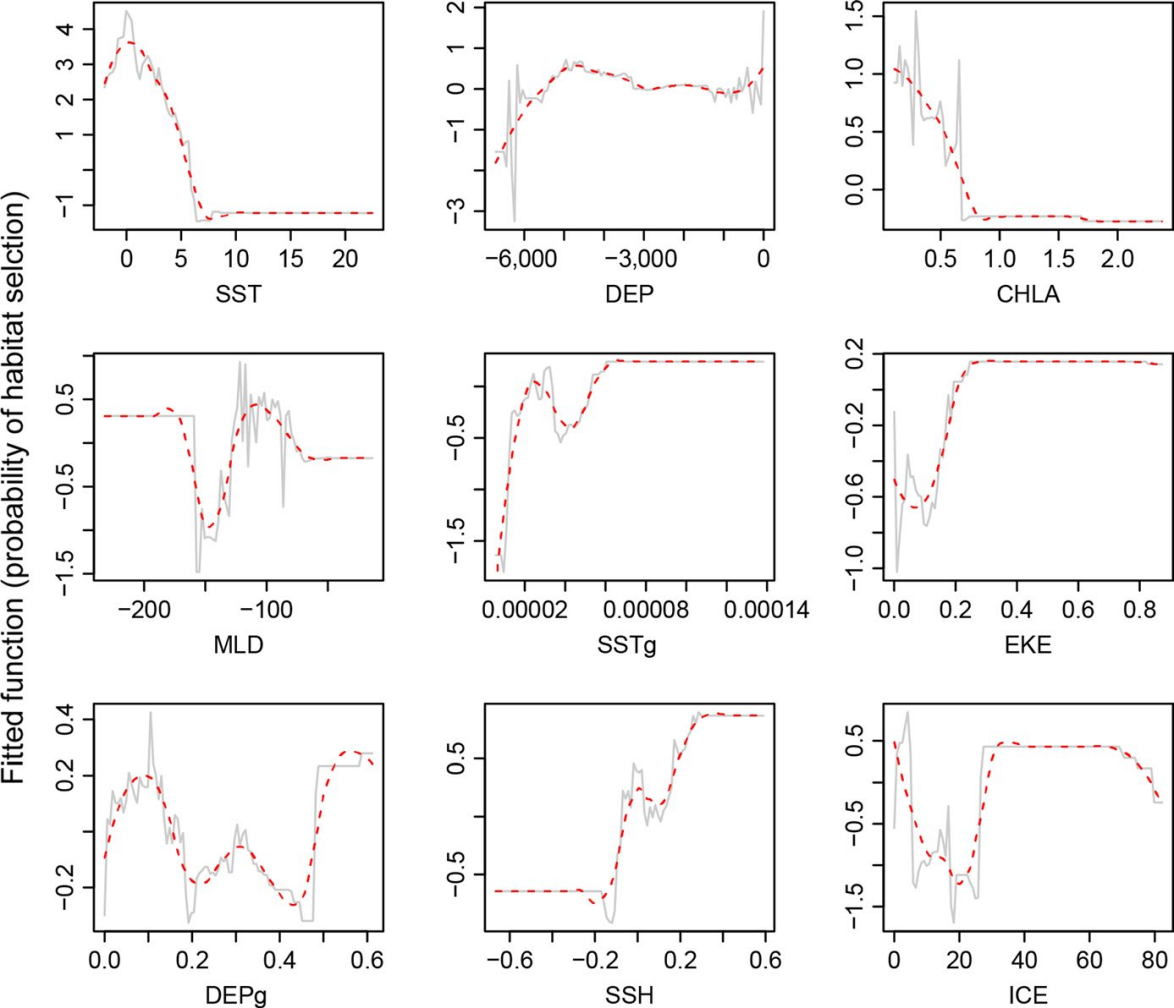
Fitted functions of nine environmental covariates explaining probability of habitat selection. Dashed red lines show smooths of the fitted functions, and plots are ordered by importance of the covariates in the final boosted regression tree model. SST, sea surface temperature (°C); DEP, depth (m); CHL, chorophyll a concentration (mg/m^3^); MLD, mixed layer depth (m); SSTg, sea surface temperature gradient (radians); EKE, eddy kinetic energy (cm^2^/s^2^); DEPg, depth gradient (radians); SSH, sea surface height anomaly (m); ICE, sea ice concentration (%)

The BRT model (mean AUC ± *SD* = 0.94 ± 0.001), identified sea surface temperature (SST) (variable relative influence = 22.0%), depth (influence = 18.5%) and chorophyll a concentration (15.4%) as the three most important predictors of habitat selection (Figure  5). Location estimates corresponded with a median SST of 2.1°C (IQR = 1.2–3.2°C) and the fitted function showed a peak probability of habitat selection at ~0° C, with the probability decreasing as SST temperature increased (Figures 6 and 7). For depth, location estimates were largely in pelagic waters (median depth = 4,003 m, IQR = 4,736–3,154 m) and the BRT predicted peak probability of habitat selection at ~5,000 to 4,000 m. The fitted function for chorophyll a showed the same pattern as SST, largely due to the high correlation between these variables (Pearson's *r *=* *.66; below our 0.7 threshold). Spatial predictions from this model emphasized the high relative habitat selection probability of the region to the southwest of Marion Island, between the Antarctic Polar Front and the southern boundary of the Antarctic Circumpolar Current.

**Figure 8:**
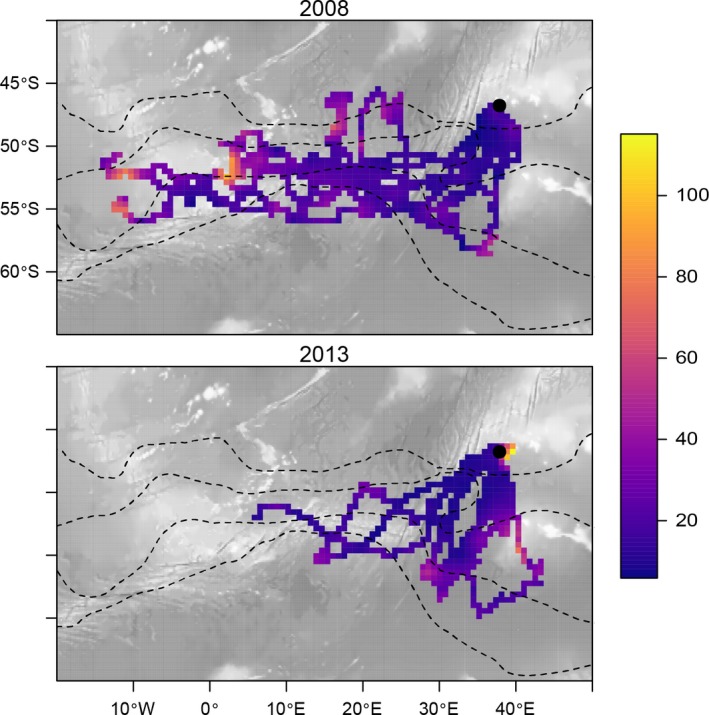
King penguin tracks depicted as mean time spent (hours) per 50 km^2^ grid cell. Dashed lines indicate the average positions of oceanographic fronts, as in Figure 1

Important foraging areas, as identified by time spent within grid cells, were spread widely with no clear distributional pattern (Figure 8). Important feeding areas straddled a wide latitudinal range with no apparent association with any fronts. An area south of Marion Island, at 52–60°S, an area west of Marion Island at about 17°W, and an area further southeast centering at 53°S and 3°E were all associated with increased time spent per grid cell. Cells directly northeast of Marion Island, including the Galieni Bank and cells adjacent to the study colonies, were also highlighted as being important in terms of foraging. This is at least partially the effect of local transit to and from colonies and orientation before departure on foraging trips. More striking than geographic areas identified as being important was the area to the west of Marion Island, associated with the Southwest Indian Ocean Ridge, where penguins spent little time per grid cell.

The time‐spent BRT model (mean ± *SD; RMSE* = 10.19 ± 0.43 hr, *R* ^2^ = 0.43 ± 0.08) predicted that SST (relative influence = 17.0%) was the most important predictor of time spent per grid cell, followed by eddy kinetic energy (16.5%) and depth (14.2%) (Figure 5). In contrast to the habitat selection model, the time‐spent model predicted the highest time spent at a SST of ~5.5°C, typical of water temperatures around Marion Island and probably largely influenced by high time‐spent values near Marion Island. Time spent per cell decreased as eddy kinetic energy increased (Figure 6).

## 4 Discussion

This study entailed the first tracking of king penguins at the Prince Edward Islands. The only previous attempt at estimating foraging range was based on a combination of foraging trip duration of breeding adults and swimming speed, but provided no information on actual distribution (Adams, 1987). With no positional data, mean maximum foraging range was crudely estimated to be about 300 km with a maximum of 900 km from the colony. Based on the current study it is, however, evident that king penguins regularly forage beyond 3,000 km from Marion Island. Based on existing literature, the previous two extreme records for foraging range of adult king penguins are 2,200 and 2,300 km from Crozet and Heard islands, respectively (Moore, Wienecke, & Robertson, 1999; Pütz, 2002). Our results therefore indicate that king penguins from Marion Island are the furthest ranging within this species recorded to date. They further demonstrate the large‐scale dispersal abilities of king penguins. At such a large scale, both meso‐ and macroscale processes are expected to be important in governing the foraging distribution.

It is clear from this study that there was a strong tendency for king penguins breeding at Marion Island to forage south and west of the Prince Edward Islands archipelago, thereby restricting the geographic extent of the processes that could be relevant in terms of concentrating prey and driving their foraging behavior. In relatively close proximity to the island, penguins generally moved in a southerly or southwesterly direction before dispersing to the west. As has been observed elsewhere (Guinet et al., 1997), some birds foraged near the pack ice but most tracks deviated westwards between about 50 and 55°S.

The behavioral contrast between king penguins at Marion Island and those breeding elsewhere in the Southern Ocean is interesting. Nowhere else has a westward displacement, against the prevailing Antarctic Circumpolar Current (ACC), characterized foraging distributions of adult king penguins. In contrast, several tracking studies on king penguins have reported a reliance on prevailing currents, arguably for rapid passage to distant foraging grounds (Baylis et al., 2015; Cotté et al., 2007). Following fasting and chick provisioning on land, it is reasonable to assume firstly that there is a high premium on energetic replenishment prior to provisioning for chicks (Kooyman et al., 1992) and secondly that the use of current flow would increase the rate of passage to prey resources and diminish energetic demands (Cotté et al., 2007; Pütz, Ingham, Smith, & Lüthi, 2002). For example, king penguins have elsewhere been shown to use southward currents to rapidly get to foraging grounds associated with the APF (Cotté et al., 2007).

Although the observed outgoing movement against the current is unique in adult king penguins, it has been reported for recently fledged individuals in the Atlantic Ocean (Pütz et al., 2014). It was proposed that this behavior could be explained by birds using olfactory cues to find areas of high productivity (Pütz et al., 2014). That king penguins forage to the west of Marion Island, despite the costs of moving against the current, suggests that they target particularly rich foraging grounds. Unlike other studies (Cotté et al., 2007), we did not see any clear spatial adjustment in relation to currents. The decrease in time spent with increasing eddy kinetic energy was furthermore surprising and suggests a relationship between this parameter and the king penguins’ prey resources.

Understanding the processes that are important in governing the distribution and dynamics of myctophids, the primary prey of king penguins (Adams & Klages, 1987; Cherel, Pütz, & Hobson, 2002), is important in order better to understand physical factors that king penguins use as cues to locate profitable prey patches. Mesoscale eddies form important feeding habitat for a number of marine top predators due to the fact that they concentrate prey resources in biologically rich areas (Cotté et al., 2007; Nel et al., 2001). Nutrient‐enriched upwelled waters in these areas stimulate phytoplankton growth leading to cascading productivity from zooplankton through to myctophids at the mesopelagic level, which king penguins and other top predators target (Hyrenbach, Veit, Weimerskirch, & Hunt, 2006; Pakhomov & McQuaid, 1996). For example, king penguins tracked from the Crozet archipelago largely foraged within mesoscale frontal zones and strong currents associated with eddies, particularly at the northern limit of the Polar Front (50–51°S) and the southern limit of the sub‐Antarctic Front (45°S) (Cotté et al., 2007). Regions of high mesoscale variability are, however, often a result of the ACC interacting with prominent bottom topography. The southwest Indian Ridge (SWIR) to the east of the Prince Edward Islands between 25 and 35°E is characterized by a number of fracture zones, resulting in the ACC being split into several branches, which give rise to enhanced eddy formation east of the ridge (Ansorge, Pakhomov, Kaehler, Lutjeharms, & Durgadoo, 2010). Our results indicate that king penguins broadly associated with sea surface height anomalies, which are characteristic of mesoscale eddies. Yet, the region to the west of Marion Island associated with the SWIR, which is characterized by mesoscale eddies (Ansorge et al., 2010; Bernard et al., 2007) and has been termed a “life support system” for marine top predators (Nel et al., 2001), was essentially a dead zone for king penguins as they rapidly traversed it on route to more distant foraging grounds.

Throughout most of their distribution, king penguins have been shown to forage in close proximity and to the south of the Polar Front within relatively cold SST waters (Guinet et al., 1997). Here Antarctic waters sink below the sub‐Antarctic waters bringing nutrients closer to the surface and it is consequently likely that myctophids aggregate relatively high up in the water column (Bost et al., 1997; Cotté et al., 2007; Pütz, 2002). Sea surface temperature was the most influential predictor of foraging distribution of adult king penguins from Marion Island, with cold waters being preferentially targeted. This is in line with previous studies that have demonstrated the importance of cold Antarctic waters for foraging king penguins. However, whereas the pack ice region has been identified as an important foraging area for king penguins from the neighboring Crozet Archipelago (Charrassin & Bost, 2001), birds from Marion Island generally did not venture this far south with only a few foraging trips potentially extending into the pack ice.

Mode of foraging and prey species that are targeted obviously influence habitat preferences. For example, a large component of the diet of chick‐rearing grey‐headed albatrosses, which target eddies on either side of the SWIR, consists of the fish *Magnisudis prionosa*, a Paralepidid, and the squid *Martialia hyadesi* (Nel et al., 2001) that cumulatively makes up <1% of the diet of king penguins at Marion Island (Adams & Klages, 1987). King penguins predominantly feed on myctophids occurring at considerable depths (Duhamel, Koubbi, & Ravier, 2000). Although the mesotrophic level is the most data deficient in this region, it is generally thought that myctophids associate with frontal systems. As found elsewhere in the Southern Indian Ocean (Bost et al., 1997), king penguins at Marion Island primarily feed on *Electrona carlsbergi,* a species that migrates within the water column in response to zooplankton prey movements (Kozlov, Shust, & Zemsky, 1991). The indication that mesoscale features to the west of Marion Island do not constitute an important foraging area is intriguing. Perhaps these mesoscale features simply do not host a large prey biomass. In accordance, Bernard and Froneman (2005) found, against expectations of enhanced biological activity, that the region to the southwest of the Prince Edward Islands had surprisingly low productivity despite prevailing eddy features, which was attributed to the origin of water making up the eddies (Strzelecki, Koslow, & Waite, 2007). Clearly a better mechanistic understanding of the interactions between the different trophic levels associated with the SWIR region is required as associated mesoscale features appear to be important for only some predatory species breeding at the Prince Edward Islands.

Results from the current study largely reflect the foraging range and habitat preferences of king penguins at Marion Island during winter. During this period, adults can spend up to 5 months at sea between chick‐provisioning events (Weimerskirch, Stahl, & Jouventin, 1992), enabling the long distance dispersal that was particularly apparent during 2008. Elsewhere, king penguins have been recorded feeding well beyond the APF in Antarctic waters during winter while feeding in close proximity to the APF during summer when young chicks demand more frequent provisioning (Charrassin & Bost, 2001; Pütz, 2002). It would be interesting, and this should be a future research priority, to obtain tracking data from king penguins from Marion Island during early summer to ascertain whether this population adheres to the general pattern observed elsewhere during the brooding phase.

## Conflict of Interest

None declared.
